# Analytical dataset of Ecuadorian cocoa shells and beans

**DOI:** 10.1016/j.dib.2018.11.129

**Published:** 2018-11-29

**Authors:** Giorgio Grillo, Luisa Boffa, Arianna Binello, Stefano Mantegna, Giancarlo Cravotto, Farid Chemat, Tatiana Dizhbite, Liga Lauberte, Galina Telysheva

**Affiliations:** a Dipartimento di Scienza e Tecnologia del Farmaco, University of Turin, 10235 Turin, Italy; b Université d’Avignon et des Pays de Vaucluse, INRA, UMR408, GREEN Team Extraction, F-84000 Avignon, France; c Latvian State Institute of Wood Chemistry, 1006 Riga, Latvia

## Abstract

Full analytical data of Ecuadorian cocoa wastes (raw shells) and beans (as benchmark), are herein reported. A detailed characterization of production residues may pave the road to a zero-waste strategy for the cocoa industry. Multiple analytical techniques have been exploited to define the composition of the matrices, among them: elemental analyses, FTIR, Py-GC/MS/FID and UHPLC-ESI-MS/MS.

Quali-quantitative data of carbohydrates, lipids, lignin, polyphenols, alkaloids and proteins have been obtained by Py-GC/MS/FID and UHPLC-ESI-MS/MS. Assignations are fully supported by literature references. The FAMEs composition of lipophilic UAE extract is also reported for sake of comparison with cocoa butter. This data collection completes a wider valorization work, “Cocoa bean shell waste valorisation; extraction from lab to pilot-scale cavitational reactors” (Grillo et al., 2018).

**Specifications table**

**Table:** 

Subject area	*Chemistry*
More specific subject area	*Extraction and Valorization*
Type of data	*Tables and figures (chromatograms, spectra and instruments)*
How data was acquired	*FTIR spectra: FTIR spectrometer (Spectrum One, PerkinElmer);*
	*CHN contents: according to EN 15104:2011 standard, elemental analyser (Vario MACRO, ELEMENTAR Analysensysteme);*
	*Carbohydrate composition: Alditol acetate procedure, GC-FID quantification (Agilent 6850 Series GC system);*
	*Py-GC/MS/FID: Frontier Lab Micro Double-shot Pyrolyser (Py-3030D), coupled to a Shimadzu 2D FID/MS gas chromatography system (MS-GC/GC–MS-2010);*
	*FAMEs composition: GC–MS qualitative analysis (Agilent Technologies 6850, Network GC System using a 5973 Network Mass Selective Detector) and GC-FID quantitative analysis (Agilent Technologies 7820A, Network GC System equipped with a FID detector);*
	*UHPLC-ESI-MS/MS: UPLC system (Acquity, Waters Corp., Singapore), coupled with a quadrupole-time of flight (Q-TOF) MS instrument (UPLC/Synapt Q-TOF MS, Waters, Milford, MA, USA) with an electrospray ionisation (ESI) source.*
Data format	Raw, analysed and formatted.
Experimental factors	*Analysed samples are composed by cocoa shells, raw or extracted by UAE. Cocoa beans were used as a benchmark.*
Experimental features	*Multiple analysis were performed for the sake of comparison between cocoa beans and residual biomass (shells) or its extract.*
Data source location	*All matrices originate from Ecuador. The Cocoa bean shells were kindly provided by Gobino S.r.l. (Turin, Italy).*
Data accessibility	*Data are reported in this article.*
Related research article	G. Grillo, L. Boffa, A. Binello, S. Mantegna, G. Cravotto, F. Chemat, T. Dizhbite, L. Lauberte, G. Telysheva,. Cocoa bean shell waste valorisation; extraction from lab to pilot-scale cavitational reactors, FOODRES-D-18–01707R1 (2018) (In Press) [1].

**Value of the data**

• Full chemical characterization of raw waste material from cocoa industry (shells).
• Spectra and chromatograms can be used as fingerprints for quick matching.
• The comparison with cocoa beans composition shed light on the potential use and exploitability of the recovered fractions.
• Reported data could pave the way to new valorization processes, providing a useful benchmark.

## 1 Data

A fingerprint of the matrices is obtained by FTIR spectra and elemental analysis. Carbohydrates composition was defined by alditol acetate protocol and by Py-GC/MS/FID. This technique was also used to quantify lipids, lignin, alkaloids, proteins and polyphenols by means of precursor identification. Definition of polyphenols was achieved by UHPLC-ESI-MS/MS analysis. Furthermore, FAMEs identification and quantification of shells lipophilic extract is reported. All data can be used for a full comparison with extracts reported by Grillo et al. [1].

## 2 Experimental design, materials and methods

### 2.1 Materials

Cocoa beans and shells from Ecuador were kindly provided by Gobino S.r.l. (Turin, Italy). Shells extracts are provided according the procedure reported by Grillo et al. (see Ref. [1], Paragraph *2.3.1 US-assisted extraction*).

### 2.2 Methods

#### 2.2.1 FTIR analysis

**Fig. 1:**
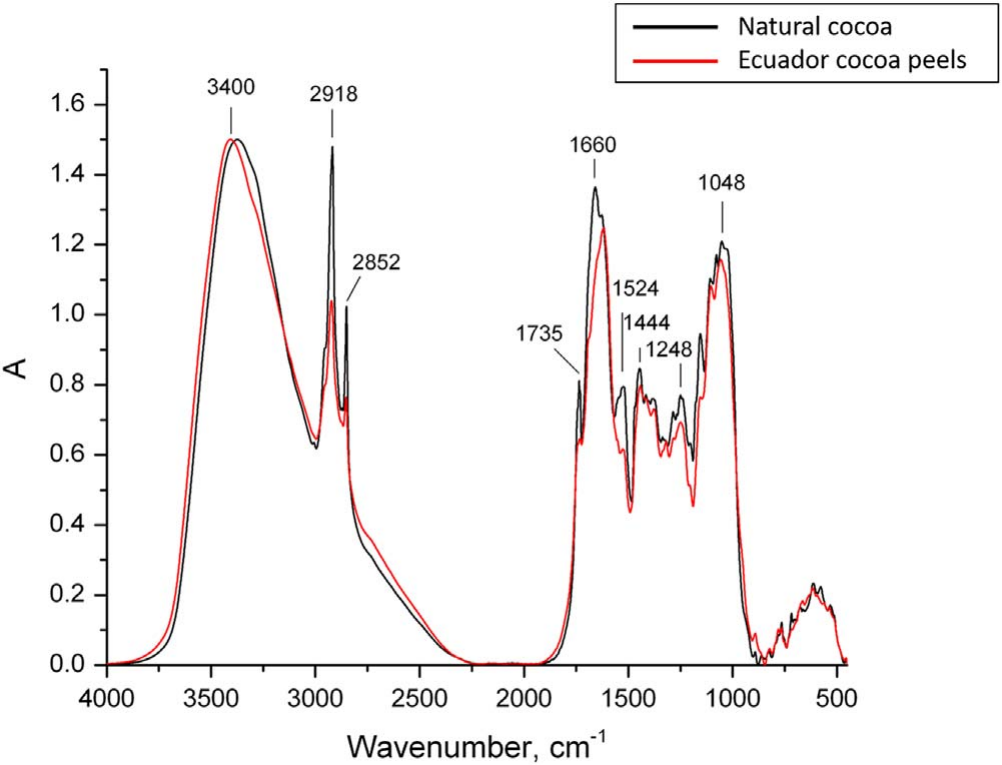
Normalized FTIR spectra of the cocoa beans (black) and shells (red).

FTIR spectra of the cocoa beans and shells (raw material) samples were recorded in KBr pellets on a Spectrum One FTIR spectrometer (PerkinElmer) in the 4000–450 cm^−1^ range (resolution 4 cm^−1^, number of scans 64). The resulting spectra (Fig. 1) were normalised to the highest absorption intensity in each spectrum (in the ca. 3400 cm^-1^ range). Bands assignments are reported in Table 1, according to wave numbers.

**Table 1:** Bands assignments in the FTIR spectra of the cocoa samples.

**Wave number (cm**^**-1**^**)**	**Assignment**
3367	-OH stretching vibration
2918, 2851	C-H stretch in CH_2_ and CH_3_ groups, mainly in lipids
1735	C=O stretch in unconjugated esters, carboxylic acids, aldehydes and ketones
1660	C=C valence deformation in fatty acid plus C=O stretch in conjugated aryl ketones
1630	amide I in proteins (C=O stretch in amide)
1549	amide II in proteins (NH_2_ deformation vibration)
1510	aromatic skeletal vibrations, mainly phenolics
1444	deformation vibration of C-H in CH_2_ and CH_3_ groups of carbohydrates
1285	C-H stretch (various)
1250	C-O valent deformation in acetyl groups
1152	C-O-C asymmetric vibration in carbohydrates and glucosides
1107 - 1028	C-C, C-OH, C-H various vibrations in carbohydrates
890 - 763	out-of-plane aromatic C-H vibrations
717	long chain C-C skeletal vibration in fatty acid

#### 2.2.2 CHN content

**Fig. 2:**
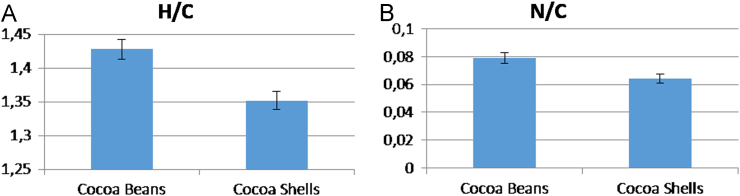
H/C (A) and N/C (B) atomic ratios for cocoa beans and shells.

C, H, N, contents in cocoa beans and shells (raw material) were measured according to the EN 15104:2011 standard using a Vario MACRO elemental analyser (ELEMENTAR Analysensysteme). Direct comparisons of H/C and N/C for the two matrices are reported in Fig. 2.

#### 2.2.3 Carbohydrate composition

Carbohydrate composition of cocoa beans and shells (raw material) was determined using an alditol acetate procedure by Blakeney, Harris, Henry and Stone, 1983 [2] after cocoa sample hydrolysis with 72% sulphuric acid. The alditol acetates were quantified by GC-FID (Agilent 6850 Series GC system) using a DB1701 column (60 m x 0.25 mm, film thickness 0.25 µm), and methyl α-*D*-glucopyranoside as the internal standard.

Results were expressed as mannose (Man), galactose (Gal), glucose (Glc), rhamnose (Rha), arabinose (Ara) and xylose (Xyl) contents (Table 2).

**Table 2:** Carbohydrates contents in cocoa samples, determined using an alditol acetate procedure.

**Cocoa Sample**	**Carbohydrates % (w/w on o.d. ash free biomass)**								
	**Rha**	**Ara**	**Xyl**	**Man**	**Gal**	**Glc**		**Total as MS**	**Total as PS**
						**Tot**	**Cellulose**		
Beans	<0.01	1.9±0.03	0.1±0.01	0.7±0.01	2.0±0.1	18.8±0.5	18.5±0.5	23.5±0.5	21.1±0.5
Shells	0.8±0.1	1.7±0.1	1.2±0.2	2.6±0.2	3.1±0.2	16.5±0.5	15.1±0.5	25.9±0.5	23.2±0.5

MS= monosaccharides, PS= polysaccharides

#### 2.2.4 Pyrolyser(Py)-GC/MS/FID analysis

Py-GC/MS/FID analysis of cocoa beans and shells (raw material) were performed using a Frontier Lab Micro Double-shot Pyrolyser Py-3030D (pyrolysis temperature 500 °C, heating rate 600 °C/s) that was directly coupled to a Shimadzu 2D FID/MS gas chromatography system MS-GC/GC–MS-2010 with a RTX-1701 capillary column (Restek, 60 m x 0.25 mm x 0.25 μm film). The injector temperature was 250 °C, the ion source 250 °C (EI 70 eV), the MS scan range m/z was 15 to 350, the carrier gas was helium (flow rate 1 mL min^-1^) and the split ratio was 1:30. The amount of sample analysed was 1.00÷2.00 mg. The oven temperature was kept at 60 °C for 1 min, increased at 6 °C/min to 270 °C and finally held at 270 °C for 10 min.

The identification of the individual compounds was performed using GC/MS chromatograms from the Library MS NIST 14, whereas the relative peak area of individual compounds was calculated using Shimadzu software on the basis of GC/FID data. The summed molar areas of the relevant peaks were normalised to 100% and the data for 5 repetitive pyrolysis experiments, at least, were averaged. Relative peak areas, calculated as percentages, for pyrolysis products of different origin were used to assess biomass sample composition (Table 3). Measurement error did not exceed 5% of the mean area value.

**Table 3:** Summary of cocoa samples Py-GC/MS/FID analysis, including GC diagnostic peaks assignments and relative contents (%) of carbohydrates (CH), lipids (Lip), fatty acids (FA), lignin (Lg) and other polyphenols (Pph), alkaloids (Alk) and proteins (Pr) derived products detected in volatiles.

**Compound/Group of compounds**	**Compounds precursors**	**Compound proportion in volatiles from analytical pyrolysis, %**	
		**Beans**	**Shells**
**Acids, Esters, Aldehydes, Ketones, Cyclopentane deriv., Furan derive., Sugars, including:**	**Carbohydrates**	**37.21**	**44.28**
acetic acid	CH	10.53	18.27
2-oxo-propanoic acid	CH	0.06	0.12
propanoic acid	CH	0.58	1.49
2-propenoic acid, methyl ester	CH	0.19	0.20
2-oxo- propanoic acid, methyl ester	CH	0.67	0.51
3-methyl- butanoic acid	CH	0.20	0.16
propanoic acid, 2-methylpropyl ester	CH	0.15	n.d.
pentanoic acid	CH	n.d.	n.d.
2-methyl-propanal	CH	1.19	0.53
2,3-butanedione	CH	1.95	2.86
3-methyl- butanal	CH	1.19	0.59
2-methyl-butanal	CH	0.95	0.69
3-methyl-3-buten-2-one	CH	0.06	n.d.
2-butenal	CH	0.06	0.00
1-hydroxy- 2-propanone	CH	7.59	7.15
2-propanone,	CH	2.51	1.33
1-(acetyloxy)-2-butanone	CH	0.13	0.10
pentanal	CH	1.67	0.86
2-cyclopenten-1-one	CH	0.78	0.80
2-methyl- 2-cyclopenten-1-one	CH	0.33	0.55
1,2-cyclopentanedione	CH	1.43	1.31
2,3-dimethyl- 2-cyclopenten-1-one	CH	n.d.	0.14
3-methyl-2-cyclopenten-1-one	CH	0.20	0.37
2-cyclopenten-1-one, 2,3-dimethyl-, isomer	CH	0.22	0.39
3-methyl-1,2-cyclopentanedione	CH	1.25	1.47
3-ethyl-2-hydroxy-2-cyclopenten-1-one	CH	0.47	0.53
2(3H)-furanone	CH	0.28	0.24
3(2H)-furanone	CH	0.48	0.33
furfural	CH	0.33	0.53
acetylfuran	CH	0.41	0.59
5-methyl-2-furancarboxaldehyde	CH	0.07	0.20
2(3H)- dihydro-furanone	CH	0.52	0.92
2(5H)-furanone	CH	0.76	0.61
isosorbide (1,4;3,6-dianhydro-*D*-glucitol)	CH	n.d.	0.43
**Phenyl and benzyl derivatives, including:**	**Lignin + Polyphenols**	**7.70**	**7.76**
methyl-benzene	Pph	0.93	0.61
ethyl-benzene,	Pph	0.33	0.45
ethenyl-benzene,	Pph	0.28	0.24
phenol	Pph, Lg	2.42	2.25
2-methyl-phenol, (o-cresol)	Pph, Lg	0.58	0.59
4-methyl- and 3-methyl-phenol, (p- & m-cresols)	Pph, Lg	2.05	1.82
3,4-dimethyl-phenol	Pph	0.26	0.24
4-ethyl-phenol	Pph	0.33	0.41
***1,2-benzenediol (tannins derivative)***	Pph	***0.15***	***0.16***
guaiacol	Lg	0.07	0.35
4-vinylguaiacol	Lg	n.d.	0.08
syringol	Lg	0.11	0.31
2,3-dihydro-benzofuran	Lg	0.19	0.24
**aliphatic compounds, including:**	**Lipids + Fatty acids**	**16.83**	**4.63**
1-nonene	Lip	0.24	n.d.
undecane	Lip	0.11	n.d.
1-undecene	Lip	0.32	n.d.
(Z)-5-undecene	Lip	0.15	0.06
(Z)-3-octen-2-ol	Lip	0.33	0.24
Dodecane	Lip	0.22	0.16
1-dodecene	Lip	0.35	0.12
1-dodecyne	Lip	0.11	n.d.
tridecane	Lip	0.22	0.12
(Z)-6-tridecene	Lip	0.35	0.12
tetradecane	Lip	0.30	0.10
1-tetradecene	Lip	0.56	0.10
3,4-dimethylcyclopentanone	Lip	0.54	0.41
pentadecane	Lip	1.49	0.59
1-pentadecene	Lip	0.26	0.06
1-hexadecene	Lip	0.69	0.18
8-heptadecene	Lip	1.10	0.16
heptadecane	Lip	1.43	0.31
(Z)-3-hexadecene	Lip	0.15	n.d.
2-hexadecanone	Lip	0.30	0.20
pentadecanoic acid, ethyl ester	FA	n.d.	0.16
octadecanoic acid, 2-propenyl ester	FA	1.95	0.16
n-hexadecanoic acid	FA	0.87	1.06
2-nonadecanone	Lip	0.19	n.d.
cyclododecanemethanol	Lip	1.28	n.d.
octadecanoic acid, 2-propenyl ester, isomer	FA	2.86	0.20
hexadecanoic acid, ethenyl ester	FA	0.45	0.08
**alkaloids derived volatiles, including:**	**Alkaloids**	**34.15**	**38.75**
1H-pyrrole, 1-methyl-	Alk	0.56	0.39
pyridine or picolinic acid	Alk	0.49	0.99
1H-pyrrole, 1-ethyl-	Alk	0.25	0.49
pyrrole	Alk	2.85	3.75
1H-pyrrole, 2-methyl-	Alk	0.47	0.24
1H-pyrrole, 2-ethyl-	Alk	0.93	0.63
1H-pyrrole, 3-ethyl-	Alk	0.06	0.03
2,5-pyrrolidinedione	Alk	0.91	0.93
**1H-purine-2,6-dione, 3,7-dihydro-1,3,7-trimethyl- (Caffeine)**	Alk	**3.44**	**4.53**
**1H-purine-2,6-dione, 3,7-dihydro-3,7-dimethyl- (Theobromine)**	Alk	**20.76**	**25.30**
indole	Alk	2.56	0.78
1H-indole, 3-methyl-	Alk	0.88	0.69
**amides and nitriles, including:**	**Lipids + Proteins**	**3.57**	**1.87**
propanenitrile	Lip, Pr	0.17	0.64
3-methyl-butanenitrile	Lip, Pr	0.27	0.28
4,4-dimethyl-3-oxopentanenitrile	Lip, Pr	n.d.	0.35
4-methyl-pentanenitrile	Lip, Pr	n.d.	0.35
tetradecanenitrile	Lip, Pr	0.46	0.25
hexadecanenitrile	Lip, Pr	0.40	n.d.
tetradecanamide	Lip, Pr	0.72	n.d.
(Z)-9-octadecenamide	Lip, Pr	0.62	n.d.
octadecanamide	Lip, Pr	0.93	n.d.

^*^n.d.- not detected

#### 2.2.5 UHPLC-ESI-MS/MS analysis of polyphenols

**Fig. 3:**
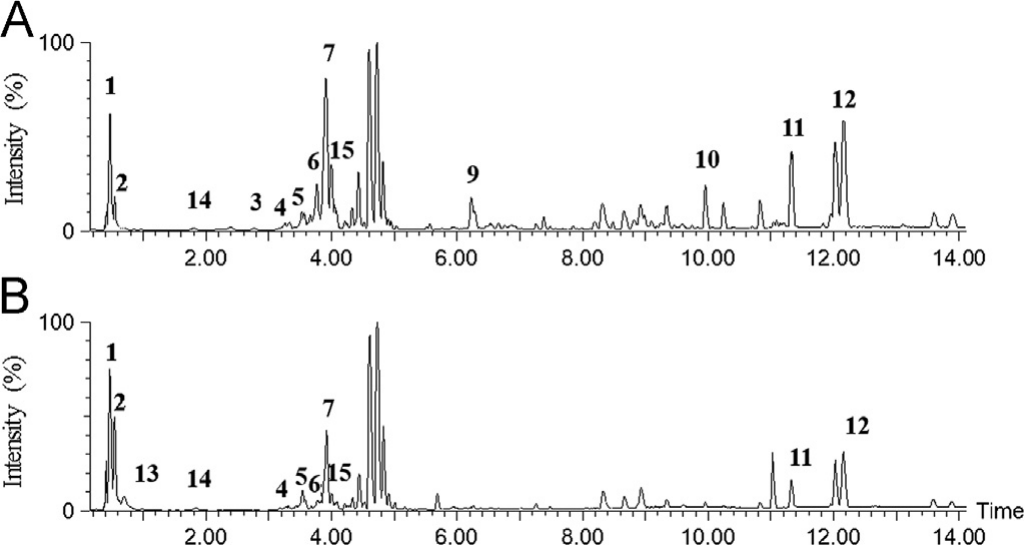
Total ion chromatogram (negative ionisation) resulting from the UHPLC-ESI-MS/MS analysis of the conventional extracts obtained from Ecuador cocoa shells: (A) 70% v/v aqueous acetone extract; (B) 80% v/v aqueous ethanol extract.

Analytical samples of raw cocoa shells were obtained from: 70% v/v aqueous acetone extract (A) and 80% v/v aqueous ethanol extract (B). All samples were dissolved in a 1:1 acetonitrile/water mixture, filtered over a nylon filter (0.45 μm pore size) and analysed by UHPLC-ESI-MS/MS (Fig. 3). An Acquity UPLC system (Waters Corp., Singapore) that was coupled with a quadrupole-time of flight (Q-TOF) MS instrument (UPLC/Synapt Q-TOF MS, Waters, Milford, MA, USA) with an electrospray ionisation (ESI) source was used. A U-HPLC column (2.1 mm x 50 mm i.d., 1.7 µm, BEHC18, Waters Acquity) was used at a flow rate of 0.30 mL min^-1^. The mobile phases were water with 0.1% formic acid (A) and acetonitrile (B). The gradient program was: 0–0.5 min, 5%–5% (B); 0.5–10 min, 5%–95% (B); 10–15 min, 95%–95% (B). The injection volume was 2 μL. The major operating parameters for Q-TOF MS were set as follows: capillary voltage, 2 kV (–); cone voltage, 40 V; cone gas flow, 50 L/h; collision energy, 4 eV; source temperature, 120 °C; desolvation temperature, 350 °C; collision gas, argon; desolvation gas, nitrogen; flow rate, 600 L/h; data acquisition range, *m*/*z* 50–1.200 Da; ionisation mode, negative.

Peaks assignments has been performed by mass fragmentations (Table 4), available literature reference are shown. A composition comparison is possible, referring to UHPLC-ESI-MS/MS analysis of cocoa shells extract, reported by Grillo et al. (see Fig. 3 and Table 9 in Ref. [1], Paragraph. 3.3.2 *Extraction screening*).

**Table 4:** Polyphenols detected in the UHPLC-ESI-MS/MS analysis of the conventional extracts obtained from cocoa shells (Fig. 3).

**Compound**	**Peak Nr.**	**[M-H], Main fragments**	**Ref.**
Ggluconic acid sodium salt/glucose acid	1	195, 177, 129, 85, 75	[3]
citric acid	2	191, 111, 87	[3]
protocatechuic acid	3	153, 109, 65	[3]
procyanidin tetramer	4	1153, 577, 289	[4]
*N*-caffeoyl-*L*-aspartate derivative	5	276, 179, 131	[5]
catechin or epicatechin with a cinnamic acid side-group	6	633, 329, 305, 289, 267, 225	[6]
procyanidin dimer	7	730, 577, 289, 165	–
catechin/epicatechin derivative	8	289, 245,205,179	–
flavone/luteolin	9	329, 311, 229, 211, 171, 139, 127	[3]
hydroxybenzoic acid sugar derivative	10	299, 137	–
linoleic acid	11	279	[3]
oleic acid	12	281	–
citric acid derivative	13	191, 111, 87	[3]
coumaric acid derivative	14	163, 145	[7]
procyanidin trimer	15	865, 860, 577, 305, 289, 245	[4]

#### 2.2.6 GC analysis of fatty acid methyl esters (FAMEs)

The fatty acid composition of the lipophilic (hexane) phase derived from a ternary mixture extracts.

(see Ref. [1], Paragraph *2.3.1 US-assisted extraction*) of raw shells, was determined according to the procedure described by Bermúdez Menéndez *et al.* in 2014 [8]. GC–MS qualitative analysis were performed in an Agilent Technologies 6850 Network GC System using a 5973 Network Mass Selective Detector, a 7683B Automatic Sampler (Santa Clara, California, USA), and a capillary column (HP-5MS 5% Phenyl Methyl Siloxane, length 30 m, i.d. 0.25 mm, film thickness 0.25 μm). GC-FID quantitative analysis were performed in an Agilent Technologies 7820 A Network GC System equipped with a FID detector, using a capillary column (Mega WAX, length 30 m, i.d. 0.25 mm, film thickness 0.25 μm, Mega S.r.l., Legnano, MI, Italy) and according to the internal standard amount (methyl heptadecanoate, Me C17). All the lipophilic extracts (~10 mg) were derivatised before analysis [8].

FAMEs identification was performed by checking correspondence with C8–C24 saturated and unsaturated external standards (Sigma-Aldrich), which were prepared in solution with GC grade cyclohexane, and with Wiley7n and NIST11 GC libraries (for GC–MS analysis). All identification and quantification results are reported in Table 5, showing an overall matching with cocoa butter content profile, according to literature [9].

**Table 5:** FAMEs composition of the hexane phase from UAE extracts obtained using the ternary mixture, expressed as w/w percentage on the extract.

**FAMEs**	**w/w %**
Me myristate-C14	0.7
Me palmitate-C16	28.5
Me palmitoleate-C16:1(n-7)	0.8
Me stearate-C18	31.6
Me oleate-C18:1(cis,n-6)	32.7
Me hexadecenoate-C18:1(*cis*,n-9 o 5)	0.6
Me linoleate-C18:2(*cis*,n-6)	1.0
Me eicosanoate-C20	1.3
Me 11-eicosaenoate-C20:1(*cis*,n-9)	0.2
Me arachidonate-C20:4(*cis*,n-6)	0.1
Me 5,8,11,14,17-eicosapentaenoate-C20:5(*cis*,n-3)	0.1
Me docosanoate-C22	0.5
Me tetracosanoate-C24	0.1
Me pentacosanoate-C25	0.2
Me hexacosanoate-C26	0.3
*Total*	*98.7*
