# Supplementary material for: Analytical dataset of Ecuadorian cocoa shells and beans
Source: Data Brief. 2018 Nov 29;22:56–64. doi: 10.1016/j.dib.2018.11.129 (PMC6297061; doi:10.1016/j.dib.2018.11.129)
Supplement: Supplementary file 1 — Supplementary material [file mmc1.docx]

# Conflict of Interest

The authors declare that the research was conducted in the absence of any commercial or financial relationships that could be construed as a potential conflict of interest.
